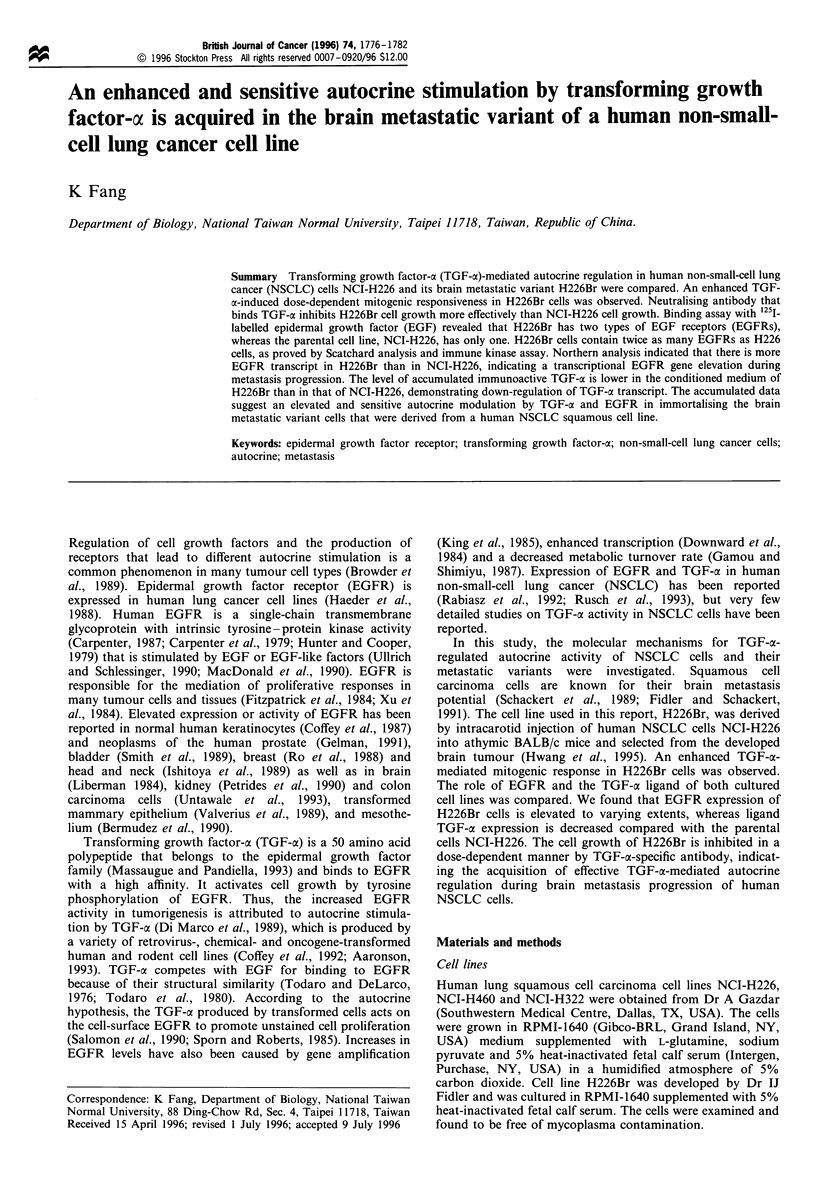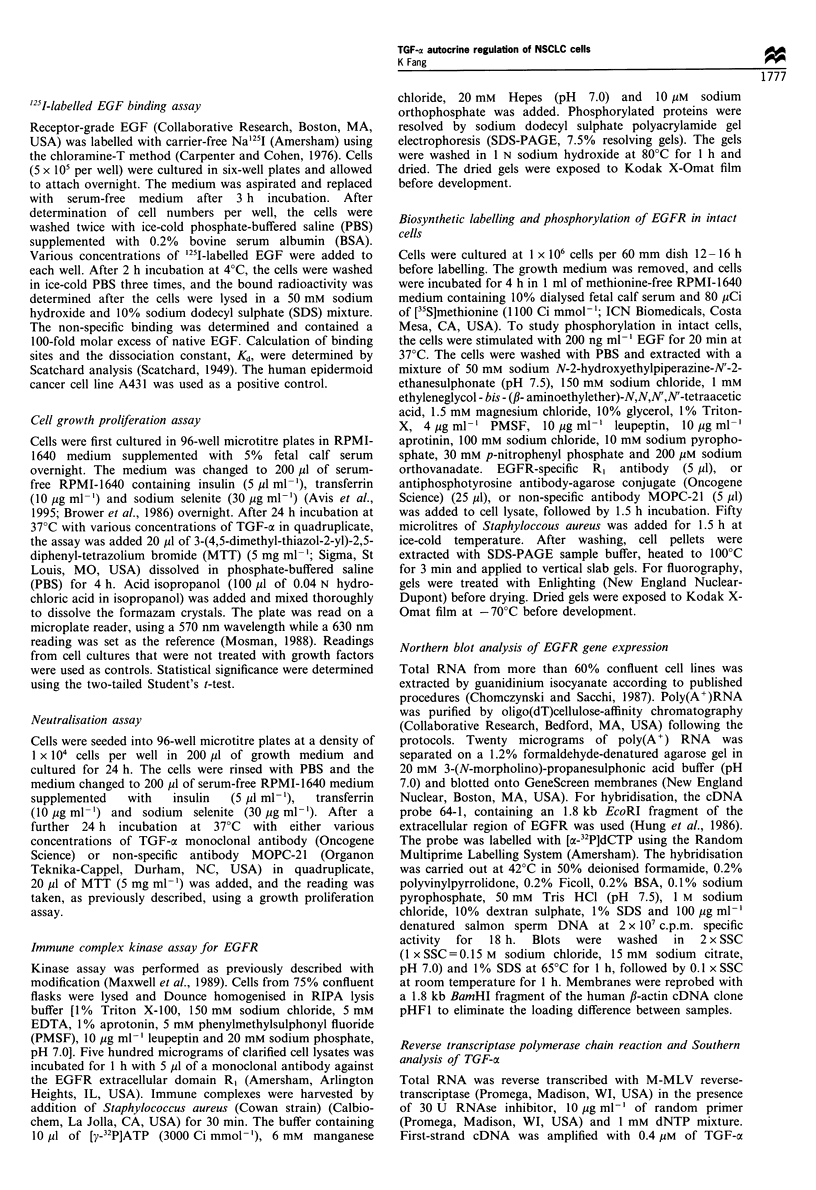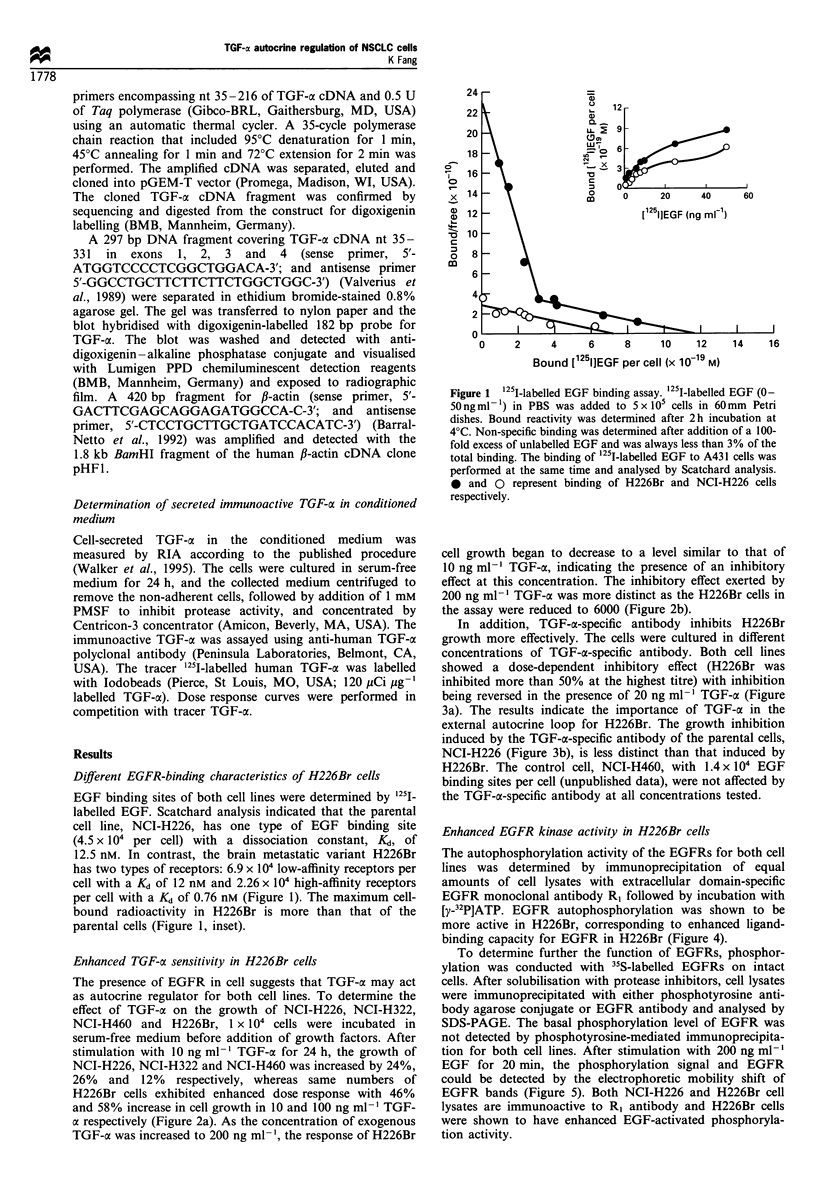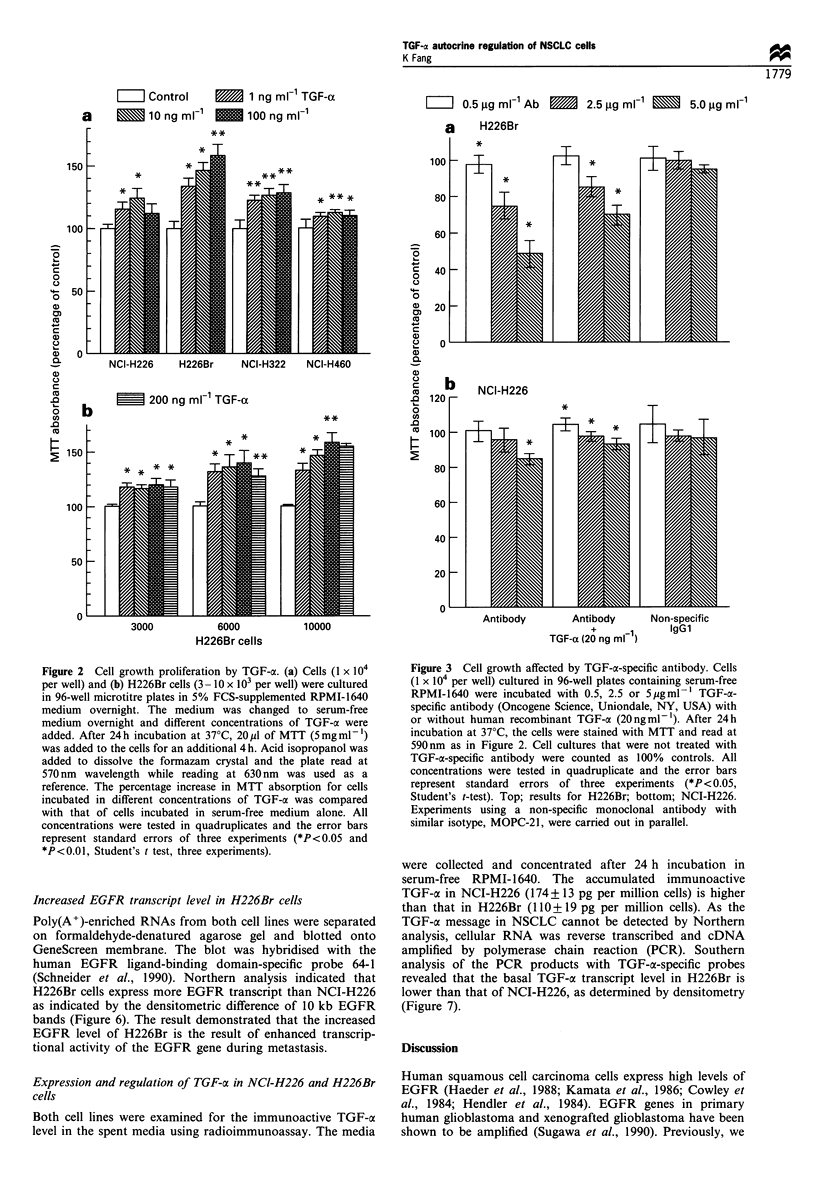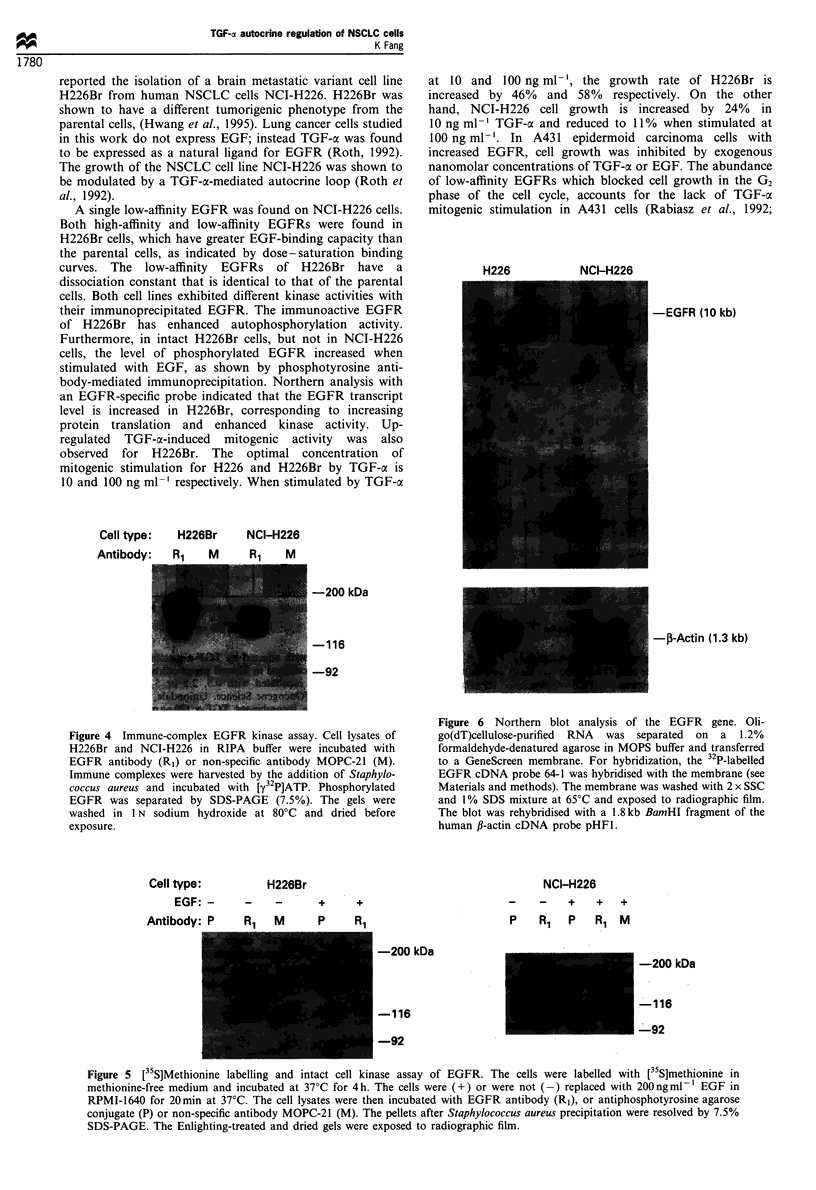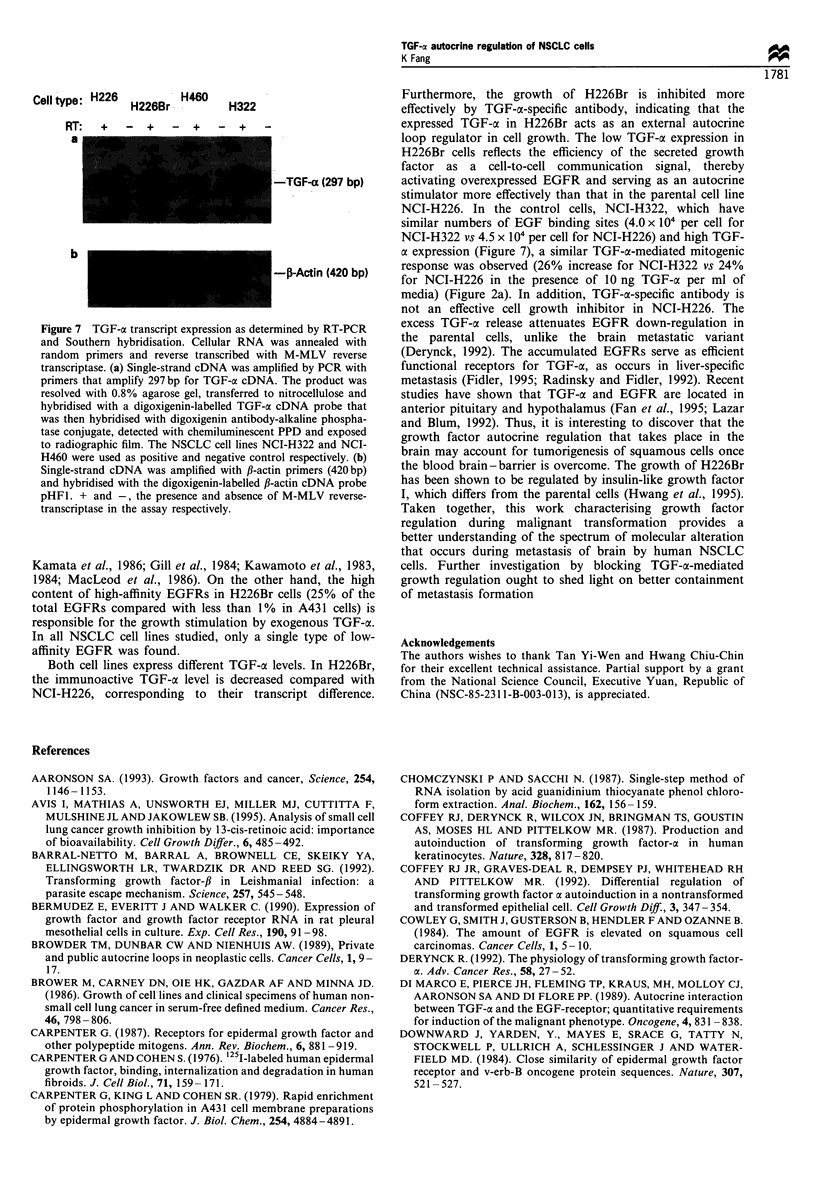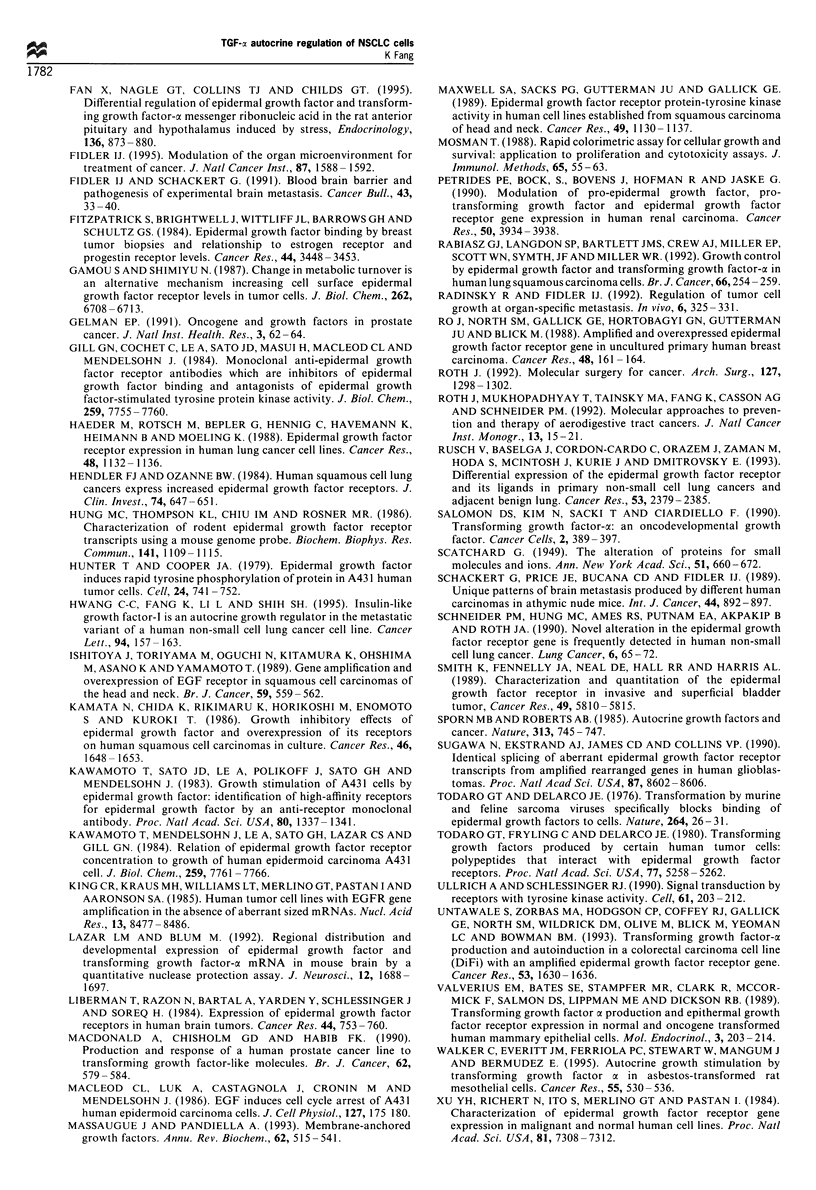# An enhanced and sensitive autocrine stimulation by transforming growth factor-alpha is acquired in the brain metastatic variant of a human non-small-cell lung cancer cell line.

**DOI:** 10.1038/bjc.1996.629

**Published:** 1996-12

**Authors:** K. Fang

**Affiliations:** Department of Biology, National Taiwan Normal University, Taipei, Republic of China.

## Abstract

**Images:**


British Journal of Cancer (1996) 74, 1776-1782
© 1996 Stockton Press All rights reserved 0007-0920/96 $12.00

### An enhanced and sensitive autocrine stimulation by transforming growth factor-α is acquired in the brain metastatic variant of a human non-small-cell lung cancer cell line

K Fang

*Department of Biology, National Taiwan Normal University, Taipei 11718, Taiwan, Republic of China.*

**Summary** Transforming growth factor-α (TGF-α)-mediated autocrine regulation in human non-small-cell lung cancer (NSCLC) cells NCI-H226 and its brain metastatic variant H226Br were compared. An enhanced TGF-α-induced dose-dependent mitogenic responsiveness in H226Br cells was observed. Neutralising antibody that binds TGF-α inhibits H226Br cell growth more effectively than NCI-H226 cell growth. Binding assay with $^{125}$I-labelled epidermal growth factor (EGF) revealed that H226Br has two types of EGF receptors (EGFRs), whereas the parental cell line, NCI-H226, has only one. H226Br cells contain twice as many EGFRs as H226 cells, as proved by Scatchard analysis and immune kinase assay. Northern analysis indicated that there is more EGFR transcript in H226Br than in NCI-H226, indicating a transcriptional EGFR gene elevation during metastasis progression. The level of accumulated immunoactive TGF-α is lower in the conditioned medium of H226Br than in that of NCI-H226, demonstrating down-regulation of TGF-α transcript. The accumulated data suggest an elevated and sensitive autocrine modulation by TGF-α and EGFR in immortalising the brain metastatic variant cells that were derived from a human NSCLC squamous cell line.

**Keywords:** epidermal growth factor receptor; transforming growth factor-α; non-small-cell lung cancer cells; autocrine; metastasis

Regulation of cell growth factors and the production of receptors that lead to different autocrine stimulation is a common phenomenon in many tumour cell types (Browder *et al.*, 1989). Epidermal growth factor receptor (EGFR) is expressed in human lung cancer cell lines (Haeder *et al.*, 1988). Human EGFR is a single-chain transmembrane glycoprotein with intrinsic tyrosine-protein kinase activity (Carpenter, 1987; Carpenter *et al.*, 1979; Hunter and Cooper, 1979) that is stimulated by EGF or EGF-like factors (Ullrich and Schlessinger, 1990; MacDonald *et al.*, 1990). EGFR is responsible for the mediation of proliferative responses in many tumour cells and tissues (Fitzpatrick *et al.*, 1984; Xu *et al.*, 1984). Elevated expression or activity of EGFR has been reported in normal human keratinocytes (Coffey *et al.*, 1987) and neoplasms of the human prostate (Gelman, 1991), bladder (Smith *et al.*, 1989), breast (Ro *et al.*, 1988) and head and neck (Ishitoya *et al.*, 1989) as well as in brain (Liberman 1984), kidney (Petrides *et al.*, 1990) and colon carcinoma cells (Untawale *et al.*, 1993), transformed mammary epithelium (Valverius *et al.*, 1989), and mesothelium (Bermudez *et al.*, 1990).

Transforming growth factor-α (TGF-α) is a 50 amino acid polypeptide that belongs to the epidermal growth factor family (Massaugue and Pandiella, 1993) and binds to EGFR with a high affinity. It activates cell growth by tyrosine phosphorylation of EGFR. Thus, the increased EGFR activity in tumorigenesis is attributed to autocrine stimulation by TGF-α (Di Marco *et al.*, 1989), which is produced by a variety of retrovirus-, chemical- and oncogene-transformed human and rodent cell lines (Coffey *et al.*, 1992; Aaronson, 1993). TGF-α competes with EGF for binding to EGFR because of their structural similarity (Todaro and DeLarco, 1976; Todaro *et al.*, 1980). According to the autocrine hypothesis, the TGF-α produced by transformed cells acts on the cell-surface EGFR to promote unstained cell proliferation (Salomon *et al.*, 1990; Sporn and Roberts, 1985). Increases in EGFR levels have also been caused by gene amplification (King *et al.*, 1985), enhanced transcription (Downward *et al.*, 1984) and a decreased metabolic turnover rate (Gamou and Shimiyu, 1987). Expression of EGFR and TGF-α in human non-small-cell lung cancer (NSCLC) has been reported (Rabiasz *et al.*, 1992; Rusch *et al.*, 1993), but very few detailed studies on TGF-α activity in NSCLC cells have been reported.

In this study, the molecular mechanisms for TGF-α-regulated autocrine activity of NSCLC cells and their metastatic variants were investigated. Squamous cell carcinoma cells are known for their brain metastasis potential (Schackert *et al.*, 1989; Fidler and Schackert, 1991). The cell line used in this report, H226Br, was derived by intracarotid injection of human NSCLC cells NCI-H226 into athymic BALB/c mice and selected from the developed brain tumour (Hwang *et al.*, 1995). An enhanced TGF-α-mediated mitogenic response in H226Br cells was observed. The role of EGFR and the TGF-α ligand of both cultured cell lines was compared. We found that EGFR expression of H226Br cells is elevated to varying extents, whereas ligand TGF-α expression is decreased compared with the parental cells NCI-H226. The cell growth of H226Br is inhibited in a dose-dependent manner by TGF-α-specific antibody, indicating the acquisition of effective TGF-α-mediated autocrine regulation during brain metastasis progression of human NSCLC cells.

### Materials and methods

### *Cell lines*

Human lung squamous cell carcinoma cell lines NCI-H226, NCI-H460 and NCI-H322 were obtained from Dr A Gazdar (Southwestern Medical Centre, Dallas, TX, USA). The cells were grown in RPMI-1640 (Gibco-BRL, Grand Island, NY, USA) medium supplemented with L-glutamine, sodium pyruvate and 5% heat-inactivated fetal calf serum (Intergen, Purchase, NY, USA) in a humidified atmosphere of 5% carbon dioxide. Cell line H226Br was developed by Dr IJ Fidler and was cultured in RPMI-1640 supplemented with 5% heat-inactivated fetal calf serum. The cells were examined and found to be free of mycoplasma contamination.

Correspondence: K Fang, Department of Biology, National Taiwan Normal University, 88 Ding-Chow Rd, Sec. 4, Taipei 11718, Taiwan
Received 15 April 1996; revised 1 July 1996; accepted 9 July 1996

*$^{125}$I-labelled EGF binding assay*

Receptor-grade EGF (Collaborative Research, Boston, MA, USA) was labelled with carrier-free Na$^{125}$I (Amersham) using the chloramine-T method (Carpenter and Cohen, 1976). Cells ($5 \times 10^5$ per well) were cultured in six-well plates and allowed to attach overnight. The medium was aspirated and replaced with serum-free medium after 3 h incubation. After determination of cell numbers per well, the cells were washed twice with ice-cold phosphate-buffered saline (PBS) supplemented with 0.2% bovine serum albumin (BSA). Various concentrations of $^{125}$I-labelled EGF were added to each well. After 2 h incubation at 4°C, the cells were washed in ice-cold PBS three times, and the bound radioactivity was determined after the cells were lysed in a 50 mM sodium hydroxide and 10% sodium dodecyl sulphate (SDS) mixture. The non-specific binding was determined and contained a 100-fold molar excess of native EGF. Calculation of binding sites and the dissociation constant, $K_d$, were determined by Scatchard analysis (Scatchard, 1949). The human epidermoid cancer cell line A431 was used as a positive control.

*Cell growth proliferation assay*

Cells were first cultured in 96-well microtitre plates in RPMI-1640 medium supplemented with 5% fetal calf serum overnight. The medium was changed to 200 μl of serum-free RPMI-1640 containing insulin (5 μl ml$^{-1}$), transferrin (10 μg ml$^{-1}$) and sodium selenite (30 μg ml$^{-1}$) (Avis *et al.*, 1995; Brower *et al.*, 1986) overnight. After 24 h incubation at 37°C with various concentrations of TGF-α in quadruplicate, the assay was added 20 μl of 3-(4,5-dimethyl-thiazol-2-yl)-2,5-diphenyl-tetrazolium bromide (MTT) (5 mg ml$^{-1}$; Sigma, St Louis, MO, USA) dissolved in phosphate-buffered saline (PBS) for 4 h. Acid isopropanol (100 μl of 0.04 N hydrochloric acid in isopropanol) was added and mixed thoroughly to dissolve the formazam crystals. The plate was read on a microplate reader, using a 570 nm wavelength while a 630 nm reading was set as the reference (Mosman, 1988). Readings from cell cultures that were not treated with growth factors were used as controls. Statistical significance were determined using the two-tailed Student's *t*-test.

*Neutralisation assay*

Cells were seeded into 96-well microtitre plates at a density of $1 \times 10^4$ cells per well in 200 μl of growth medium and cultured for 24 h. The cells were rinsed with PBS and the medium changed to 200 μl of serum-free RPMI-1640 medium supplemented with insulin (5 μl ml$^{-1}$), transferrin (10 μg ml$^{-1}$) and sodium selenite (30 μg ml$^{-1}$). After a further 24 h incubation at 37°C with either various concentrations of TGF-α monoclonal antibody (Oncogene Science) or non-specific antibody MOPC-21 (Organon Teknika-Cappel, Durham, NC, USA) in quadruplicate, 20 μl of MTT (5 mg ml$^{-1}$) was added, and the reading was taken, as previously described, using a growth proliferation assay.

*Immune complex kinase assay for EGFR*

Kinase assay was performed as previously described with modification (Maxwell *et al.*, 1989). Cells from 75% confluent flasks were lysed and Dounce homogenised in RIPA lysis buffer [1% Triton X-100, 150 mM sodium chloride, 5 mM EDTA, 1% aprotonin, 5 mM phenylmethylsulphonyl fluoride (PMSF), 10 μg ml$^{-1}$ leupeptin and 20 mM sodium phosphate, pH 7.0]. Five hundred micrograms of clarified cell lysates was incubated for 1 h with 5 μl of a monoclonal antibody against the EGFR extracellular domain $R_1$ (Amersham, Arlington Heights, IL, USA). Immune complexes were harvested by addition of *Staphylococcus aureus* (Cowan strain) (Calbiochem, La Jolla, CA, USA) for 30 min. The buffer containing 10 μl of [γ-$^{32}$P]ATP (3000 Ci mmol$^{-1}$), 6 mM manganese chloride, 20 mM Hepes (pH 7.0) and 10 μM sodium orthophosphate was added. Phosphorylated proteins were resolved by sodium dodecyl sulphate polyacrylamide gel electrophoresis (SDS-PAGE, 7.5% resolving gels). The gels were washed in 1 N sodium hydroxide at 80°C for 1 h and dried. The dried gels were exposed to Kodak X-Omat film before development.

*Biosynthetic labelling and phosphorylation of EGFR in intact cells*

Cells were cultured at $1 \times 10^6$ cells per 60 mm dish 12–16 h before labelling. The growth medium was removed, and cells were incubated for 4 h in 1 ml of methionine-free RPMI-1640 medium containing 10% dialysed fetal calf serum and 80 μCi of [$^{35}$S]methionine (1100 Ci mmol$^{-1}$; ICN Biomedicals, Costa Mesa, CA, USA). To study phosphorylation in intact cells, the cells were stimulated with 200 ng ml$^{-1}$ EGF for 20 min at 37°C. The cells were washed with PBS and extracted with a mixture of 50 mM sodium *N*-2-hydroxyethylpiperazine-*N'*-2-ethanesulphonate (pH 7.5), 150 mM sodium chloride, 1 mM ethyleneglycol-*bis*-(β-aminoethylether)-*N,N,N',N'*-tetraacetic acid, 1.5 mM magnesium chloride, 10% glycerol, 1% Triton-X, 4 μg ml$^{-1}$ PMSF, 10 μg ml$^{-1}$ leupeptin, 10 μg ml$^{-1}$ aprotinin, 100 mM sodium chloride, 10 mM sodium pyrophosphate, 30 mM *p*-nitrophenyl phosphate and 200 μM sodium orthovanadate. EGFR-specific $R_1$ antibody (5 μl), or antiphosphotyrosine antibody-agarose conjugate (Oncogene Science) (25 μl), or non-specific antibody MOPC-21 (5 μl) was added to cell lysate, followed by 1.5 h incubation. Fifty microlitres of *Staphyloccous aureus* was added for 1.5 h at ice-cold temperature. After washing, cell pellets were extracted with SDS-PAGE sample buffer, heated to 100°C for 3 min and applied to vertical slab gels. For fluorography, gels were treated with Enlighting (New England Nuclear-Dupont) before drying. Dried gels were exposed to Kodak X-Omat film at −70°C before development.

*Northern blot analysis of EGFR gene expression*

Total RNA from more than 60% confluent cell lines was extracted by guanidinium isocyanate according to published procedures (Chomczynski and Sacchi, 1987). Poly(A$^+$)RNA was purified by oligo(dT)cellulose-affinity chromatography (Collaborative Research, Bedford, MA, USA) following the protocols. Twenty micrograms of poly(A$^+$) RNA was separated on a 1.2% formaldehyde-denatured agarose gel in 20 mM 3-(*N*-morpholino)-propanesulphonic acid buffer (pH 7.0) and blotted onto GeneScreen membranes (New England Nuclear, Boston, MA, USA). For hybridisation, the cDNA probe 64-1, containing an 1.8 kb *Eco*RI fragment of the extracellular region of EGFR was used (Hung *et al.*, 1986). The probe was labelled with [α-$^{32}$P]dCTP using the Random Multiprime Labelling System (Amersham). The hybridisation was carried out at 42°C in 50% deionised formamide, 0.2% polyvinylpyrrolidone, 0.2% Ficoll, 0.2% BSA, 0.1% sodium pyrophosphate, 50 mM Tris HCl (pH 7.5), 1 M sodium chloride, 10% dextran sulphate, 1% SDS and 100 μg ml$^{-1}$ denatured salmon sperm DNA at $2 \times 10^7$ c.p.m. specific activity for 18 h. Blots were washed in $2 \times$ SSC ($1 \times$ SSC = 0.15 M sodium chloride, 15 mM sodium citrate, pH 7.0) and 1% SDS at 65°C for 1 h, followed by $0.1 \times$ SSC at room temperature for 1 h. Membranes were reprobed with a 1.8 kb *Bam*HI fragment of the human β-actin cDNA clone pHF1 to eliminate the loading difference between samples.

*Reverse transcriptase polymerase chain reaction and Southern analysis of TGF-α*

Total RNA was reverse transcribed with M-MLV reverse-transcriptase (Promega, Madison, WI, USA) in the presence of 30 U RNAse inhibitor, 10 μg ml$^{-1}$ of random primer (Promega, Madison, WI, USA) and 1 mM dNTP mixture. First-strand cDNA was amplified with 0.4 μM of TGF-α

primers encompassing nt 35–216 of TGF-α cDNA and 0.5 U of *Taq* polymerase (Gibco-BRL, Gaithersburg, MD, USA) using an automatic thermal cycler. A 35-cycle polymerase chain reaction that included 95°C denaturation for 1 min, 45°C annealing for 1 min and 72°C extension for 2 min was performed. The amplified cDNA was separated, eluted and cloned into pGEM-T vector (Promega, Madison, WI, USA). The cloned TGF-α cDNA fragment was confirmed by sequencing and digested from the construct for digoxigenin labelling (BMB, Mannheim, Germany).

A 297 bp DNA fragment covering TGF-α cDNA nt 35–331 in exons 1, 2, 3 and 4 (sense primer, 5′-ATGGTCCCCTCGGCTGGACA-3′; and antisense primer 5′-GGCCTGCTTCTTCTTCTGGCTGGC-3′) (Valverius *et al.*, 1989) were separated in ethidium bromide-stained 0.8% agarose gel. The gel was transferred to nylon paper and the blot hybridised with digoxigenin-labelled 182 bp probe for TGF-α. The blot was washed and detected with anti-digoxigenin–alkaline phosphatase conjugate and visualised with Lumigen PPD chemiluminescent detection reagents (BMB, Mannheim, Germany) and exposed to radiographic film. A 420 bp fragment for β-actin (sense primer, 5′-GACTTCGAGCAGGAGATGGCCA-C-3′; and antisense primer, 5′-CTCCTGCTTGCTGATCCACATC-3′) (Barral-Netto *et al.*, 1992) was amplified and detected with the 1.8 kb *Bam*HI fragment of the human β-actin cDNA clone pHF1.

*Determination of secreted immunoactive TGF-α in conditioned medium*

Cell-secreted TGF-α in the conditioned medium was measured by RIA according to the published procedure (Walker *et al.*, 1995). The cells were cultured in serum-free medium for 24 h, and the collected medium centrifuged to remove the non-adherent cells, followed by addition of 1 mM PMSF to inhibit protease activity, and concentrated by Centricon-3 concentrator (Amicon, Beverly, MA, USA). The immunoactive TGF-α was assayed using anti-human TGF-α polyclonal antibody (Peninsula Laboratories, Belmont, CA, USA). The tracer $^{125}$I-labelled human TGF-α was labelled with Iodobeads (Pierce, St Louis, MO, USA; 120 μCi μg$^{-1}$ labelled TGF-α). Dose response curves were performed in competition with tracer TGF-α.

### Results

*Different EGFR-binding characteristics of H226Br cells*

EGF binding sites of both cell lines were determined by $^{125}$I-labelled EGF. Scatchard analysis indicated that the parental cell line, NCI-H226, has one type of EGF binding site ($4.5 \times 10^4$ per cell) with a dissociation constant, $K_d$, of 12.5 nM. In contrast, the brain metastatic variant H226Br has two types of receptors: $6.9 \times 10^4$ low-affinity receptors per cell with a $K_d$ of 12 nM and $2.26 \times 10^4$ high-affinity receptors per cell with a $K_d$ of 0.76 nM (Figure 1). The maximum cell-bound radioactivity in H226Br is more than that of the parental cells (Figure 1, inset).

*Enhanced TGF-α sensitivity in H226Br cells*

The presence of EGFR in cell suggests that TGF-α may act as autocrine regulator for both cell lines. To determine the effect of TGF-α on the growth of NCI-H226, NCI-H322, NCI-H460 and H226Br, $1 \times 10^4$ cells were incubated in serum-free medium before addition of growth factors. After stimulation with 10 ng ml$^{-1}$ TGF-α for 24 h, the growth of NCI-H226, NCI-H322 and NCI-H460 was increased by 24%, 26% and 12% respectively, whereas same numbers of H226Br cells exhibited enhanced dose response with 46% and 58% increase in cell growth in 10 and 100 ng ml$^{-1}$ TGF-α respectively (Figure 2a). As the concentration of exogenous TGF-α was increased to 200 ng ml$^{-1}$, the response of H226Br

**Figure 1** $^{125}$I-labelled EGF binding assay. $^{125}$I-labelled EGF (0–50 ng ml$^{-1}$) in PBS was added to $5 \times 10^5$ cells in 60 mm Petri dishes. Bound reactivity was determined after 2 h incubation at 4°C. Non-specific binding was determined after addition of a 100-fold excess of unlabelled EGF and was always less than 3% of the total binding. The binding of $^{125}$I-labelled EGF to A431 cells was performed at the same time and analysed by Scatchard analysis. ● and ○ represent binding of H226Br and NCI-H226 cells respectively.

cell growth began to decrease to a level similar to that of 10 ng ml$^{-1}$ TGF-α, indicating the presence of an inhibitory effect at this concentration. The inhibitory effect exerted by 200 ng ml$^{-1}$ TGF-α was more distinct as the H226Br cells in the assay were reduced to 6000 (Figure 2b).

In addition, TGF-α-specific antibody inhibits H226Br growth more effectively. The cells were cultured in different concentrations of TGF-α-specific antibody. Both cell lines showed a dose-dependent inhibitory effect (H226Br was inhibited more than 50% at the highest titre) with inhibition being reversed in the presence of 20 ng ml$^{-1}$ TGF-α (Figure 3a). The results indicate the importance of TGF-α in the external autocrine loop for H226Br. The growth inhibition induced by the TGF-α-specific antibody of the parental cells, NCI-H226 (Figure 3b), is less distinct than that induced by H226Br. The control cell, NCI-H460, with $1.4 \times 10^4$ EGF binding sites per cell (unpublished data), were not affected by the TGF-α-specific antibody at all concentrations tested.

*Enhanced EGFR kinase activity in H226Br cells*

The autophosphorylation activity of the EGFRs for both cell lines was determined by immunoprecipitation of equal amounts of cell lysates with extracellular domain-specific EGFR monoclonal antibody $R_1$ followed by incubation with [γ-$^{32}$P]ATP. EGFR autophosphorylation was shown to be more active in H226Br, corresponding to enhanced ligand-binding capacity for EGFR in H226Br (Figure 4).

To determine further the function of EGFRs, phosphorylation was conducted with $^{35}$S-labelled EGFRs on intact cells. After solubilisation with protease inhibitors, cell lysates were immunoprecipitated with either phosphotyrosine antibody agarose conjugate or EGFR antibody and analysed by SDS-PAGE. The basal phosphorylation level of EGFR was not detected by phosphotyrosine-mediated immunoprecipitation for both cell lines. After stimulation with 200 ng ml$^{-1}$ EGF for 20 min, the phosphorylation signal and EGFR could be detected by the electrophoretic mobility shift of EGFR bands (Figure 5). Both NCI-H226 and H226Br cell lysates are immunoactive to $R_1$ antibody and H226Br cells were shown to have enhanced EGF-activated phosphorylation activity.

**Figure 2** Cell growth proliferation by TGF-α. (**a**) Cells ($1 \times 10^4$ per well) and (**b**) H226Br cells ($3-10 \times 10^3$ per well) were cultured in 96-well microtitre plates in 5% FCS-supplemented RPMI-1640 medium overnight. The medium was changed to serum-free medium overnight and different concentrations of TGF-α were added. After 24 h incubation at 37°C, 20 μl of MTT (5 mg ml$^{-1}$) was added to the cells for an additional 4 h. Acid isopropanol was added to dissolve the formazam crystal and the plate read at 570 nm wavelength while reading at 630 nm was used as a reference. The percentage increase in MTT absorption for cells incubated in different concentrations of TGF-α was compared with that of cells incubated in serum-free medium alone. All concentrations were tested in quadruplicates and the error bars represent standard errors of three experiments (*$P<0.05$ and *$P<0.01$, Student's *t* test, three experiments).

**Figure 3** Cell growth affected by TGF-α-specific antibody. Cells ($1 \times 10^4$ per well) cultured in 96-well plates containing serum-free RPMI-1640 were incubated with 0.5, 2.5 or 5 μg ml$^{-1}$ TGF-α-specific antibody (Oncogene Science, Uniondale, NY, USA) with or without human recombinant TGF-α (20 ng ml$^{-1}$). After 24 h incubation at 37°C, the cells were stained with MTT and read at 590 nm as in Figure 2. Cell cultures that were not treated with TGF-α-specific antibody were counted as 100% controls. All concentrations were tested in quadruplicate and the error bars represent standard errors of three experiments (*$P<0.05$, Student's *t*-test). Top; results for H226Br; bottom; NCI-H226. Experiments using a non-specific monoclonal antibody with similar isotype, MOPC-21, were carried out in parallel.

*Increased EGFR transcript level in H226Br cells*

Poly(A$^+$)-enriched RNAs from both cell lines were separated on formaldehyde-denatured agarose gel and blotted onto GeneScreen membrane. The blot was hybridised with the human EGFR ligand-binding domain-specific probe 64-1 (Schneider *et al.*, 1990). Northern analysis indicated that H226Br cells express more EGFR transcript than NCI-H226 as indicated by the densitometric difference of 10 kb EGFR bands (Figure 6). The result demonstrated that the increased EGFR level of H226Br is the result of enhanced transcriptional activity of the EGFR gene during metastasis.

*Expression and regulation of TGF-α in NCl-H226 and H226Br cells*

Both cell lines were examined for the immunoactive TGF-α level in the spent media using radioimmunoassay. The media were collected and concentrated after 24 h incubation in serum-free RPMI-1640. The accumulated immunoactive TGF-α in NCI-H226 (174 ± 13 pg per million cells) is higher than that in H226Br (110 ± 19 pg per million cells). As the TGF-α message in NSCLC cannot be detected by Northern analysis, cellular RNA was reverse transcribed and cDNA amplified by polymerase chain reaction (PCR). Southern analysis of the PCR products with TGF-α-specific probes revealed that the basal TGF-α transcript level in H226Br is lower than that of NCI-H226, as determined by densitometry (Figure 7).

### Discussion

Human squamous cell carcinoma cells express high levels of EGFR (Haeder *et al.*, 1988; Kamata *et al.*, 1986; Cowley *et al.*, 1984; Hendler *et al.*, 1984). EGFR genes in primary human glioblastoma and xenografted glioblastoma have been shown to be amplified (Sugawa *et al.*, 1990). Previously, we reported the isolation of a brain metastatic variant cell line H226Br from human NSCLC cells NCI-H226. H226Br was shown to have a different tumorigenic phenotype from the parental cells, (Hwang *et al.*, 1995). Lung cancer cells studied in this work do not express EGF; instead TGF-α was found to be expressed as a natural ligand for EGFR (Roth, 1992). The growth of the NSCLC cell line NCI-H226 was shown to be modulated by a TGF-α-mediated autocrine loop (Roth *et al.*, 1992).

A single low-affinity EGFR was found on NCI-H226 cells. Both high-affinity and low-affinity EGFRs were found in H226Br cells, which have greater EGF-binding capacity than the parental cells, as indicated by dose–saturation binding curves. The low-affinity EGFRs of H226Br have a dissociation constant that is identical to that of the parental cells. Both cell lines exhibited different kinase activities with their immunoprecipitated EGFR. The immunoactive EGFR of H226Br has enhanced autophosphorylation activity. Furthermore, in intact H226Br cells, but not in NCI-H226 cells, the level of phosphorylated EGFR increased when stimulated with EGF, as shown by phosphotyrosine antibody-mediated immunoprecipitation. Northern analysis with an EGFR-specific probe indicated that the EGFR transcript level is increased in H226Br, corresponding to increasing protein translation and enhanced kinase activity. Up-regulated TGF-α-induced mitogenic activity was also observed for H226Br. The optimal concentration of mitogenic stimulation for H226 and H226Br by TGF-α is 10 and 100 ng $ml^{-1}$ respectively. When stimulated by TGF-α at 10 and 100 ng $ml^{-1}$, the growth rate of H226Br is increased by 46% and 58% respectively. On the other hand, NCI-H226 cell growth is increased by 24% in 10 ng $ml^{-1}$ TGF-α and reduced to 11% when stimulated at 100 ng $ml^{-1}$. In A431 epidermoid carcinoma cells with increased EGFR, cell growth was inhibited by exogenous nanomolar concentrations of TGF-α or EGF. The abundance of low-affinity EGFRs which blocked cell growth in the $G_2$ phase of the cell cycle, accounts for the lack of TGF-α mitogenic stimulation in A431 cells (Rabiasz *et al.*, 1992;

**Figure 4** Immune-complex EGFR kinase assay. Cell lysates of H226Br and NCI-H226 in RIPA buffer were incubated with EGFR antibody ($R_1$) or non-specific antibody MOPC-21 (M). Immune complexes were harvested by the addition of *Staphylococcus aureus* and incubated with [$\gamma^{32}$P]ATP. Phosphorylated EGFR was separated by SDS-PAGE (7.5%). The gels were washed in 1 N sodium hydroxide at 80°C and dried before exposure.

**Figure 6** Northern blot analysis of the EGFR gene. Oligo(dT)cellulose-purified RNA was separated on a 1.2% formaldehyde-denatured agarose in MOPS buffer and transferred to a GeneScreen membrane. For hybridization, the $^{32}$P-labelled EGFR cDNA probe 64-1 was hybridised with the membrane (see Materials and methods). The membrane was washed with 2 × SSC and 1% SDS mixture at 65°C and exposed to radiographic film. The blot was rehybridised with a 1.8 kb *Bam*HI fragment of the human β-actin cDNA probe pHF1.

**Figure 5** [$^{35}$S]Methionine labelling and intact cell kinase assay of EGFR. The cells were labelled with [$^{35}$S]methionine in methionine-free medium and incubated at 37°C for 4 h. The cells were (+) or were not (−) replaced with 200 ng $ml^{-1}$ EGF in RPMI-1640 for 20 min at 37°C. The cell lysates were then incubated with EGFR antibody ($R_1$), or antiphosphotyrosine agarose conjugate (P) or non-specific antibody MOPC-21 (M). The pellets after *Staphylococcus aureus* precipitation were resolved by 7.5% SDS-PAGE. The Enlighting-treated and dried gels were exposed to radiographic film.

**Figure 7** TGF-α transcript expression as determined by RT-PCR and Southern hybridisation. Cellular RNA was annealed with random primers and reverse transcribed with M-MLV reverse transcriptase. (**a**) Single-strand cDNA was amplified by PCR with primers that amplify 297 bp for TGF-α cDNA. The product was resolved with 0.8% agarose gel, transferred to nitrocellulose and hybridised with a digoxigenin-labelled TGF-α cDNA probe that was then hybridised with digoxigenin antibody-alkaline phosphatase conjugate, detected with chemiluminescent PPD and exposed to radiographic film. The NSCLC cell lines NCI-H322 and NCI-H460 were used as positive and negative control respectively. (**b**) Single-strand cDNA was amplified with β-actin primers (420 bp) and hybridised with the digoxigenin-labelled β-actin cDNA probe pHF1. + and −, the presence and absence of M-MLV reverse-transcriptase in the assay respectively.

Kamata *et al.*, 1986; Gill *et al.*, 1984; Kawamoto *et al.*, 1983, 1984; MacLeod *et al.*, 1986). On the other hand, the high content of high-affinity EGFRs in H226Br cells (25% of the total EGFRs compared with less than 1% in A431 cells) is responsible for the growth stimulation by exogenous TGF-α. In all NSCLC cell lines studied, only a single type of low-affinity EGFR was found.

Both cell lines express different TGF-α levels. In H226Br, the immunoactive TGF-α level is decreased compared with NCI-H226, corresponding to their transcript difference. Furthermore, the growth of H226Br is inhibited more effectively by TGF-α-specific antibody, indicating that the expressed TGF-α in H226Br acts as an external autocrine loop regulator in cell growth. The low TGF-α expression in H226Br cells reflects the efficiency of the secreted growth factor as a cell-to-cell communication signal, thereby activating overexpressed EGFR and serving as an autocrine stimulator more effectively than that in the parental cell line NCI-H226. In the control cells, NCI-H322, which have similar numbers of EGF binding sites ($4.0 \times 10^4$ per cell for NCI-H322 *vs* $4.5 \times 10^4$ per cell for NCI-H226) and high TGF-α expression (Figure 7), a similar TGF-α-mediated mitogenic response was observed (26% increase for NCI-H322 *vs* 24% for NCI-H226 in the presence of 10 ng TGF-α per ml of media) (Figure 2a). In addition, TGF-α-specific antibody is not an effective cell growth inhibitor in NCI-H226. The excess TGF-α release attenuates EGFR down-regulation in the parental cells, unlike the brain metastatic variant (Derynck, 1992). The accumulated EGFRs serve as efficient functional receptors for TGF-α, as occurs in liver-specific metastasis (Fidler, 1995; Radinsky and Fidler, 1992). Recent studies have shown that TGF-α and EGFR are located in anterior pituitary and hypothalamus (Fan *et al.*, 1995; Lazar and Blum, 1992). Thus, it is interesting to discover that the growth factor autocrine regulation that takes place in the brain may account for tumorigenesis of squamous cells once the blood brain–barrier is overcome. The growth of H226Br has been shown to be regulated by insulin-like growth factor I, which differs from the parental cells (Hwang *et al.*, 1995). Taken together, this work characterising growth factor regulation during malignant transformation provides a better understanding of the spectrum of molecular alteration that occurs during metastasis of brain by human NSCLC cells. Further investigation by blocking TGF-α-mediated growth regulation ought to shed light on better containment of metastasis formation

**Acknowledgements**
The authors wishes to thank Tan Yi-Wen and Hwang Chiu-Chin for their excellent technical assistance. Partial support by a grant from the National Science Council, Executive Yuan, Republic of China (NSC-85-2311-B-003-013), is appreciated.

### References

AARONSON SA. (1993). Growth factors and cancer, *Science*, **254**, 1146–1153.

AVIS I, MATHIAS A, UNSWORTH EJ, MILLER MJ, CUTTITTA F, MULSHINE JL AND JAKOWLEW SB. (1995). Analysis of small cell lung cancer growth inhibition by 13-cis-retinoic acid: importance of bioavailability. *Cell Growth Differ.*, **6**, 485–492.

BARRAL-NETTO M, BARRAL A, BROWNELL CE, SKEIKY YA, ELLINGSWORTH LR, TWARDZIK DR AND REED SG. (1992). Transforming growth factor-β in Leishmanial infection: a parasite escape mechanism. *Science*, **257**, 545–548.

BERMUDEZ E, EVERITT J AND WALKER C. (1990). Expression of growth factor and growth factor receptor RNA in rat pleural mesothelial cells in culture. *Exp. Cell Res.*, **190**, 91–98.

BROWDER TM, DUNBAR CW AND NIENHUIS AW. (1989), Private and public autocrine loops in neoplastic cells. *Cancer Cells*, **1**, 9–17.

BROWER M, CARNEY DN, OIE HK, GAZDAR AF AND MINNA JD. (1986). Growth of cell lines and clinical specimens of human non-small cell lung cancer in serum-free defined medium. *Cancer Res.*, **46**, 798–806.

CARPENTER G. (1987). Receptors for epidermal growth factor and other polypeptide mitogens. *Ann. Rev. Biochem.*, **6**, 881–919.

CARPENTER G AND COHEN S. (1976). $^{125}$I-labeled human epidermal growth factor, binding, internalization and degradation in human fibroids. *J. Cell Biol.*, **71**, 159–171.

CARPENTER G, KING L AND COHEN SR. (1979). Rapid enrichment of protein phosphorylation in A431 cell membrane preparations by epidermal growth factor. *J. Biol. Chem.*, **254**, 4884–4891.

CHOMCZYNSKI P AND SACCHI N. (1987). Single-step method of RNA isolation by acid guanidinium thiocyanate phenol chloroform extraction. *Anal. Biochem.*, **162**, 156–159.

COFFEY RJ, DERYNCK R, WILCOX JN, BRINGMAN TS, GOUSTIN AS, MOSES HL AND PITTELKOW MR. (1987). Production and autoinduction of transforming growth factor-α in human keratinocytes. *Nature*, **328**, 817–820.

COFFEY RJ JR, GRAVES-DEAL R, DEMPSEY PJ, WHITEHEAD RH AND PITTELKOW MR. (1992). Differential regulation of transforming growth factor α autoinduction in a nontransformed and transformed epithelial cell. *Cell Growth Diff.*, **3**, 347–354.

COWLEY G, SMITH J, GUSTERSON B, HENDLER F AND OZANNE B. (1984). The amount of EGFR is elevated on squamous cell carcinomas. *Cancer Cells*, **1**, 5–10.

DERYNCK R. (1992). The physiology of transforming growth factor-α. *Adv. Cancer Res.*, **58**, 27–52.

DI MARCO E, PIERCE JH, FLEMING TP, KRAUS, MH, MOLLOY CJ, AARONSON SA AND DI FLORE PP. (1989). Autocrine interaction between TGF-α and the EGF-receptor; quantitative requirements for induction of the malignant phenotype. *Oncogene*, **4**, 831–838.

DOWNWARD J, YARDEN, Y., MAYES E, SRACE G, TATTY N, STOCKWELL P, ULLRICH A, SCHLESSINGER J AND WATERFIELD MD. (1984). Close similarity of epidermal growth factor receptor and v-erb-B oncogene protein sequences. *Nature*, **307**, 521–527.

FAN X, NAGLE GT, COLLINS TJ AND CHILDS GT. (1995). Differential regulation of epidermal growth factor and transforming growth factor-α messenger ribonucleic acid in the rat anterior pituitary and hypothalamus induced by stress, *Endocrinology*, **136**, 873-880.

FIDLER IJ. (1995). Modulation of the organ microenvironment for treatment of cancer. *J. Natl Cancer Inst.*, **87**, 1588-1592.

FIDLER IJ AND SCHACKERT G. (1991). Blood brain barrier and pathogenesis of experimental brain metastasis. *Cancer Bull.*, **43**, 33-40.

FITZPATRICK S, BRIGHTWELL J, WITTLIFF JL, BARROWS GH AND SCHULTZ GS. (1984). Epidermal growth factor binding by breast tumor biopsies and relationship to estrogen receptor and progestin receptor levels. *Cancer Res.*, **44**, 3448-3453.

GAMOU S AND SHIMIYU N. (1987). Change in metabolic turnover is an alternative mechanism increasing cell surface epidermal growth factor receptor levels in tumor cells. *J. Biol. Chem.*, **262**, 6708-6713.

GELMAN EP. (1991). Oncogene and growth factors in prostate cancer. *J. Natl Inst. Health. Res.*, **3**, 62-64.

GILL GN, COCHET C, LE A, SATO JD, MASUI H, MACLEOD CL AND MENDELSOHN J. (1984). Monoclonal anti-epidermal growth factor receptor antibodies which are inhibitors of epidermal growth factor binding and antagonists of epidermal growth factor-stimulated tyrosine protein kinase activity. *J. Biol. Chem.*, **259**, 7755-7760.

HAEDER M, ROTSCH M, BEPLER G, HENNIG C, HAVEMANN K, HEIMANN B AND MOELING K. (1988). Epidermal growth factor receptor expression in human lung cancer cell lines. *Cancer Res.*, **48**, 1132-1136.

HENDLER FJ AND OZANNE BW. (1984). Human squamous cell lung cancers express increased epidermal growth factor receptors. *J. Clin. Invest.*, **74**, 647-651.

HUNG MC, THOMPSON KL, CHIU IM AND ROSNER MR. (1986). Characterization of rodent epidermal growth factor receptor transcripts using a mouse genome probe. *Biochem. Biophys. Res. Commun.*, **141**, 1109-1115.

HUNTER T AND COOPER JA. (1979). Epidermal growth factor induces rapid tyrosine phosphorylation of protein in A431 human tumor cells. *Cell*, **24**, 741-752.

HWANG C-C, FANG K, LI L AND SHIH SH. (1995). Insulin-like growth factor-I is an autocrine growth regulator in the metastatic variant of a human non-small cell lung cancer cell line. *Cancer Lett.*, **94**, 157-163.

ISHITOYA J, TORIYAMA M, OGUCHI N, KITAMURA K, OHSHIMA M, ASANO K AND YAMAMOTO T. (1989). Gene amplification and overexpression of EGF receptor in squamous cell carcinomas of the head and neck. *Br. J. Cancer*, **59**, 559-562.

KAMATA N, CHIDA K, RIKIMARU K, HORIKOSHI M, ENOMOTO S AND KUROKI T. (1986). Growth inhibitory effects of epidermal growth factor and overexpression of its receptors on human squamous cell carcinomas in culture. *Cancer Res.*, **46**, 1648-1653.

KAWAMOTO T, SATO JD, LE A, POLIKOFF J, SATO GH AND MENDELSOHN J. (1983). Growth stimulation of A431 cells by epidermal growth factor: identification of high-affinity receptors for epidermal growth factor by an anti-receptor monoclonal antibody. *Proc. Natl Acad. Sci. USA*, **80**, 1337-1341.

KAWAMOTO T, MENDELSOHN J, LE A, SATO GH, LAZAR CS AND GILL GN. (1984). Relation of epidermal growth factor receptor concentration to growth of human epidermoid carcinoma A431 cell. *J. Biol. Chem.*, **259**, 7761-7766.

KING CR, KRAUS MH, WILLIAMS LT, MERLINO GT, PASTAN I AND AARONSON SA. (1985). Human tumor cell lines with EGFR gene amplification in the absence of aberrant sized mRNAs. *Nucl. Acid Res.*, **13**, 8477-8486.

LAZAR LM AND BLUM M. (1992). Regional distribution and developmental expression of epidermal growth factor and transforming growth factor-α mRNA in mouse brain by a quantitative nuclease protection assay. *J. Neurosci.*, **12**, 1688-1697.

LIBERMAN T, RAZON N, BARTAL A, YARDEN Y, SCHLESSINGER J AND SOREQ H. (1984). Expression of epidermal growth factor receptors in human brain tumors. *Cancer Res.* **44**, 753-760.

MACDONALD A, CHISHOLM GD AND HABIB FK. (1990). Production and response of a human prostate cancer line to transforming growth factor-like molecules. *Br. J. Cancer*, **62**, 579-584.

MACLEOD CL, LUK A, CASTAGNOLA J, CRONIN M AND MENDELSOHN J. (1986). EGF induces cell cycle arrest of A431 human epidermoid carcinoma cells. *J. Cell Physiol.*, **127**, 175 180.

MASSAUGUE J AND PANDIELLA A. (1993). Membrane-anchored growth factors. *Annu. Rev. Biochem.*, **62**, 515-541.

MAXWELL SA, SACKS PG, GUTTERMAN JU AND GALLICK GE. (1989). Epidermal growth factor receptor protein-tyrosine kinase activity in human cell lines established from squamous carcinoma of head and neck. *Cancer Res.*, **49**, 1130-1137.

MOSMAN T. (1988). Rapid colorimetric assay for cellular growth and survival: application to proliferation and cytotoxicity assays. *J. Immunol. Methods*, **65**, 55-63.

PETRIDES PE, BOCK, S., BOVENS J, HOFMAN R AND JASKE G. (1990). Modulation of pro-epidermal growth factor, pro-transforming growth factor and epidermal growth factor receptor gene expression in human renal carcinoma. *Cancer Res.*, **50**, 3934-3938.

RABIASZ GJ, LANGDON SP, BARTLETT JMS, CREW AJ, MILLER EP, SCOTT WN, SYMTH, JF AND MILLER WR. (1992). Growth control by epidermal growth factor and transforming growth factor-α in human lung squamous carcinoma cells. *Br. J. Cancer*, **66**, 254-259.

RADINSKY R AND FIDLER IJ. (1992). Regulation of tumor cell growth at organ-specific metastasis. *In vivo*, **6**, 325-331.

RO J, NORTH SM, GALLICK GE, HORTOBAGYI GN, GUTTERMAN JU AND BLICK M. (1988). Amplified and overexpressed epidermal growth factor receptor gene in uncultured primary human breast carcinoma. *Cancer Res.*, **48**, 161-164.

ROTH J. (1992). Molecular surgery for cancer. *Arch. Surg.*, **127**, 1298-1302.

ROTH J, MUKHOPADHYAY T, TAINSKY MA, FANG K, CASSON AG AND SCHNEIDER PM. (1992). Molecular approaches to prevention and therapy of aerodigestive tract cancers. *J. Natl Cancer Inst. Monogr.*, **13**, 15-21.

RUSCH V, BASELGA J, CORDON-CARDO C, ORAZEM J, ZAMAN M, HODA S, MCINTOSH J, KURIE J AND DMITROVSKY E. (1993). Differential expression of the epidermal growth factor receptor and its ligands in primary non-small cell lung cancers and adjacent benign lung. *Cancer Res.*, **53**, 2379-2385.

SALOMON DS, KIM N, SACKI T AND CIARDIELLO F. (1990). Transforming growth factor-α: an oncodevelopmental growth factor. *Cancer Cells*, **2**, 389-397.

SCATCHARD G. (1949). The alteration of proteins for small molecules and ions. *Ann. New York Acad. Sci.*, **51**, 660-672.

SCHACKERT G, PRICE JE, BUCANA CD AND FIDLER IJ. (1989). Unique patterns of brain metastasis produced by different human carcinomas in athymic nude mice. *Int. J. Cancer*, **44**, 892-897.

SCHNEIDER PM, HUNG MC, AMES RS, PUTNAM EA, AKPAKIP B AND ROTH JA. (1990). Novel alteration in the epidermal growth factor receptor gene is frequently detected in human non-small cell lung cancer. *Lung Cancer*, **6**, 65-72.

SMITH K, FENNELLY JA, NEAL DE, HALL RR AND HARRIS AL. (1989). Characterization and quantitation of the epidermal growth factor receptor in invasive and superficial bladder tumor, *Cancer Res.*, **49**, 5810-5815.

SPORN MB AND ROBERTS AB. (1985). Autocrine growth factors and cancer. *Nature*, **313**, 745-747.

SUGAWA N, EKSTRAND AJ, JAMES CD AND COLLINS VP. (1990). Identical splicing of aberrant epidermal growth factor receptor transcripts from amplified rearranged genes in human glioblastomas. *Proc. Natl Acad Sci. USA*, **87**, 8602-8606.

TODARO GT AND DELARCO JE. (1976). Transformation by murine and feline sarcoma viruses specifically blocks binding of epidermal growth factors to cells. *Nature*, **264**, 26-31.

TODARO GT, FRYLING C AND DELARCO JE. (1980). Transforming growth factors produced by certain human tumor cells: polypeptides that interact with epidermal growth factor receptors. *Proc. Natl Acad. Sci. USA*, **77**, 5258-5262.

ULLRICH A AND SCHLESSINGER RJ. (1990). Signal transduction by receptors with tyrosine kinase activity. *Cell*, **61**, 203-212.

UNTAWALE S, ZORBAS MA, HODGSON CP, COFFEY RJ, GALLICK GE, NORTH SM, WILDRICK DM, OLIVE M, BLICK M, YEOMAN LC AND BOWMAN BM. (1993). Transforming growth factor-α production and autoinduction in a colorectal carcinoma cell line (DiFi) with an amplified epidermal growth factor receptor gene. *Cancer Res.*, **53**, 1630-1636.

VALVERIUS EM, BATES SE, STAMPFER MR, CLARK R, MCCORMICK F, SALMON DS, LIPPMAN ME AND DICKSON RB. (1989). Transforming growth factor α production and epithermal growth factor receptor expression in normal and oncogene transformed human mammary epithelial cells. *Mol. Endocrinol.*, **3**, 203-214.

WALKER C, EVERITT JM, FERRIOLA PC, STEWART W, MANGUM J AND BERMUDEZ E. (1995). Autocrine growth stimulation by transforming growth factor α in asbestos-transformed rat mesothelial cells. *Cancer Res.*, **55**, 530-536.

XU YH, RICHERT N, ITO S, MERLINO GT AND PASTAN I. (1984). Characterization of epidermal growth factor receptor gene expression in malignant and normal human cell lines. *Proc. Natl Acad. Sci. USA*, **81**, 7308-7312.